# Thyroxine and cardiac electrophysiology—a forgotten physiological duo?

**DOI:** 10.1186/1756-6614-5-8

**Published:** 2012-08-22

**Authors:** James Ker

**Affiliations:** 1 Department of Physiology, University of Pretoria, PO Box 24318, Gesina, Pretoria 0031, South Africa

**Keywords:** Hypothyroidism, Late potentials, Sudden cardiac death

## Abstract

Thyroid hormone exerts numerous effects on the cardiovascular system. Hypothyroidism can lead to various electrocardiographic and mechanical changes in the heart and blood vessels.

The potential risk for sudden cardiac death in patients with hypothyroidism have never been properly explored. However, numerous reports of various electrocardiographic changes indicative of such a risk has been published.

In this case report the occurrence of ventricular late potentials in a case of overt hypothyroidism is described and furthermore, the disappearance of these potentials with T4 treatment alone is shown.

It is concluded that the concept that undiagnosed and/or untreated hypothyroidism poses a risk for sudden cardiac death is worth exploring.

## Introduction

Thyroid hormone has important physiological effects on the cardiovascular system [1]. Cardiovascular effects of hypothyroidism can include electrocardiographic changes, such as bradycardia, right bundle branch block, flattened or inverted T waves, QRS prolongation and even torsades de pointes ventricular arrhythmia [2].

Mechanical cardiovascular effects of hypothyroidism include a remarkable increase in peripheral vascular resistance [3], an increase in arterial stiffness [4], an impairment in left ventricular diastolic function as characterized by a slowing of myocardial relaxation and impaired early ventricular filling [5] and pericardial effusion [6].

Currently, there is an interesting electrocardiographic contrast between thyrotoxicosis and hypothyroidism [2]: In thyrotoxicosis atrial tachyarrhythmias are common and ventricular arrhythmias are rare. However, in hypothyroidism QT interval prolongation and ever QT dispersion can occur and lead to ventricular arrhythmias, such as torsade de pointes ventricular tachycardia which can be resolved with T4 treatment alone [2,7].

In this case report it is shown that severe, primary hypothyroidism can present with an abnormal signal averaged electrocardiogram and that this can be corrected with T4 treatment alone. To date this is the only report of primary hypothyroidism presenting with an abnormal signal-averaged electrocardiogram corrected with T4 treatment alone.

## Case report

**Figure 1:**
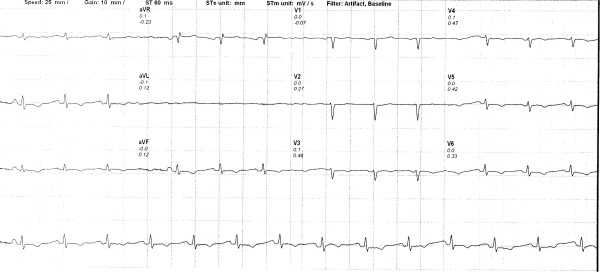
12lead surface electrocardiogram.

A 66 year old Caucasian woman was referred for a cardiovascular examination due to an abnormal electrocardiogram, taken by her primary care physician after complaining of tiredness. The 12 lead, surface electrocardiogram revealed the presence of low QRS voltages and flattened and inverted T waves, all findings suggestive of and compatible with hypothyroidism (Figure 1).

At no stage did the patient experience any symptoms suggestive of hypothyroidism or any other disease.

The serum TSH (thyroid stimulating hormone) measured 76.11 mIU/L and was indicative of severe, primary hypothyroidism (normal range 0.27 – 4.20 mIU/L).

No other pathology was found. Specifically no secondary hyperlipidemia was present and the serum glucose level was normal. No classical signs of hypothyroidism were present. Thyroid ultrasonography revealed a small and hypoechogenic thyroid gland with the typical appearance of advanced Hashimoto thyroiditis with no nodules present.

**Figure 2:**
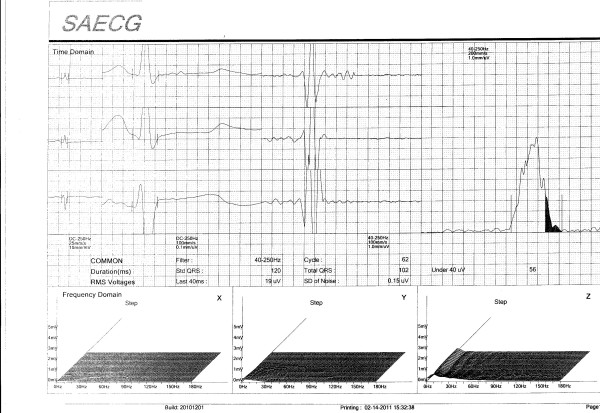
Signal averaged electrocardiogram before thyroxine replacement.

A signal-averaged electrocardiogram (SAECG) was done and this was clearly abnormal (Figure 2) with the root-mean square voltage of the terminal 40 ms (RMS 40) measuring 19uV and the duration of low-amplitude signal < 40uV (LASd) measuring 56 ms.

**Figure 3:**
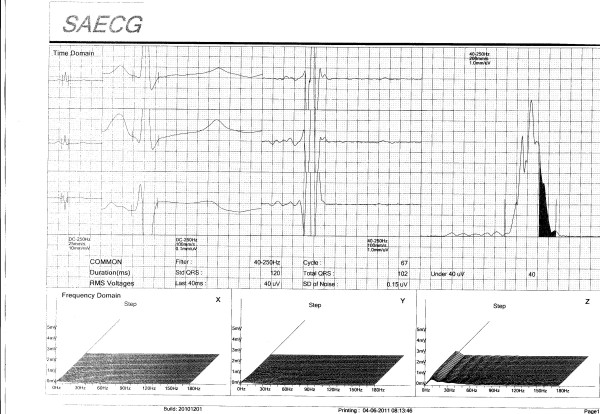
Signal averaged electrocardiogram after thyroxine replacement.

The patient was treated with 100 ug of T4 daily and the TSH level after two months of therapy measured 2.34 mIU/L and the signal-averaged electrocardiogram was repeated (Figure 3). The RMS 40 now measured 40 uV and the LASd measured 40 ms. The measured parameters of the SAECG thus changed from clearly abnormal into the normal range (see Figure 2 and Figure 3). During the first two weeks of therapy the dose of T4 was started at 50 ug daily and this was increased to 100 ug daily thereafter.

The patient remains well with a normal signal-averaged electrocardiogram after one year of clinical follow-up.

## Discussion

Three aspects of this case merit discussion. Firstly, this is the first case report in the literature which describes the presence of ventricular late potentials in the myocardium of a patient with overt hypothyroidism. The signal-averaged electrocardiogram is a technique used to detect the presence of ventricular late potentials [8]. Ventricular late potentials correspond to areas in the ventricular myocardium where there is slowed conduction velocity and these cause delayed ventricular activation [8]. These ventricular late potentials indicate an increased risk for the occurrence of ventricular arrhythmias [8]. These observed ventricular late potentials disappeared in this patient after T4 treatment alone.

Secondly, other parameters indicating an increased ventricular arrhythmic risk has been described in hypothyroidism [9-11]. An increase in the QTc interval have been described in hypothyroidism and that this increase is directly related to the severity of hypothyroidism [9]. TSH levels have also been shown to be directly related to QT prolongation and QT dispersion [10]. QT dispersion is the interlead variability of the QT interval on the surface ECG that reflects regional variations in myocardial repolarization and an increased QT dispersion has been found to be strongly associated with an increase in ventricular arrhythmias and sudden cardiac death [2]. Lastly, an improvement in heart rate variability have also been documented in treated hypothyroidism [2].

Thirdly, hypothyroidism can affect cardiac structure [11-13]. These structural effects manifests clinically in hypothyroidism as an increase in myocardial echoreflectivity [13,14], perhaps an explanation for the observed electrocardiographic abnormalities observed in hypothyroidism?

## Conclusions

In conclusion, a case is presented showing the presence of cardiac late potentials in a patient with overt hypothyroidism. Disappearance of these late potentials with only T4 treatment is shown. It is proposed that undiagnosed and/or untreated hypothyroidism poses a threat of sudden cardiac death and that this concept is worthwhile to be studied in a proper randomized trial.
